# A case report of androgen receptor inhibitor therapy in recurrent high-grade serous ovarian cancer

**DOI:** 10.18632/oncotarget.27809

**Published:** 2020-11-17

**Authors:** Sewanti Limaye, Prashant Kumar, Ramya Pragya, Janani Sambath, Darshana Patil, Ajay Srinivasan, Sachin Apurva, Navin Srivastava, Sanket Patil, Revati Patil, Vineet Datta, Dadasaheb Akolkar, Rajan Datar

**Affiliations:** ^1^Kokilaben Dhirubhai Ambani Hospital and Medical Research Institute, Mumbai, Maharashtra, India; ^2^Datar Cancer Genetics Limited, Nasik, Maharashtra, India; ^3^Institute of Bioinformatics, International Technology Park, Bangalore, Karnataka, India; ^4^Manipal Academy of Higher Education, Manipal, Karnataka, India

**Keywords:** high grade serous ovarian cancer, androgen receptors, bicalutamide

## Abstract

Ovarian cancer is common gynaecological malignancy and a leading cause of death among women. Despite the advances in treatment strategies, majority of patients present with recurrence after first- or second-line treatment. Targeted therapy that has proven to be effective in other advanced or metastatic solid tumors have also demonstrated its efficacy in ovarian cancer. Recent studies have shown that the androgen receptor (AR) signalling is involved in pathogenicity and progression of cancer. Current observations suggest AR could be a potential target in managing the disease. In this case report we present a patient with high grade serous ovarian cancer (HGSOC) with multiple relapses with excellent disease control on AR inhibition with bicalutamide.

## INTRODUCTION

Ovarian cancer is the third most common gynaecological malignancy and is the fifth leading cause of death in women. Due to lack of proper screening and early diagnostics, 70% of the patients are diagnosed with advanced stage ovarian cancer. The standard treatment for ovarian cancer includes surgery and combination treatment with carboplatin and paclitaxel. Many patients respond to the first line therapy; however 60–70% and 80–85% of women with residual disease < 1 cm and large-volume residual disease show recurrence [1]. Lack of proper molecular stratification and resistance to the conventional chemotherapy account for higher mortality.

The presence of hormone receptors such as androgen receptors (AR), progesterone receptors (PR) and estrogen receptors (ER) has been positively correlated with progression of many cancers including ovarian cancer [2–4]. The ER, PR and AR pathways are involved in regulating the signalling pathways such as cell-proliferation, apoptosis, epithelial to mesenchymal transition, cell migration and invasion. The role of AR in female includes muscle strength and volume, erythropoietin production, bone formation/growth, and differentiation/maturation of bone marrow stem cells. The role of AR in tumorigenesis and tumor progression of breast and ovarian cancer of women has been previously reported [5, 6]. It has been reported that the expression of AR in ovarian cancer is more related to the cancer subtype than the FIGO staging [7]. Toledo *et al.* reported that the serous ovarian cancer have more prevalence of AR expression than the non-serous ovarian cancer [8]. AR directed therapy is an active area of interest in management of different cancers and much effort is ongoing to target AR and AR-related pathways. Current evidences from the reported studies show selection of appropriate patients based on AR expression, AR polymorphism, and activity of AR downstream targets could be useful to optimize the clinical outcome [9, 10].

In this study, we report a case of recurrent high-grade serous ovarian cancer patient who had a durable response to AR targeted therapy designed based on Encyclopedic Tumor Analysis (ETA) (Exacta^®^) [11] which is an integrative, multi-analyte test and includes molecular analysis of comprehensive gene expression, DNA mutation profiling, chemosensitivity assay, immunohistochemistry and immunocytochemistry on tumor tissue and blood.

## CASE PRESENTATION

A 57-year-old female with a prior history of hypertension, was diagnosed with Stage IIIC high-grade serous ovarian carcinoma (HGSOC) nearly 5 years ago. The patient received neo-adjuvant chemotherapy with paclitaxel and carboplatin every 3 weeks for 3 cycles. A repeat scan done post 3 cycles showed partial response to treatment and patient underwent a cytoreductive surgery at an outside center. The surgical pathology was consistent with Stage IIIC disease noted earlier with lack of good response to carboplatin and paclitaxel given neoadjuvantly. Hence treatment was switched to gemcitabine, cisplatin and bevacizumab during adjuvant therapy for 3 cycles. A repeat scan done post completion of treatment showed complete resolution of disease.

**Figure 1:**
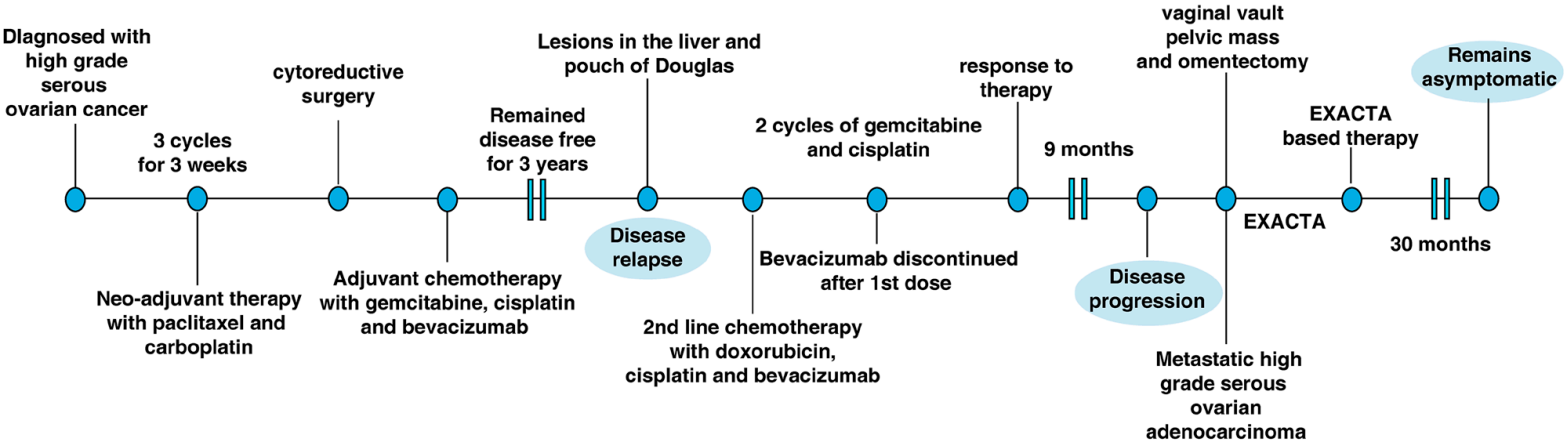
The clinical time line of the 57-year old female presented with high-grade serous ovarian cancer.

The patient remained disease free but had a relapse with the PET scan showing FDG avid lesions in the liver and pouch of Douglas after 3 years. The patient was started on second-line chemotherapy with gemcitabine, cisplatin and bevacizumab. However, bevacizumab was discontinued after first dose as the patient developed internal bleeding in the operative bed. Next two cycles of chemotherapy were continued with gemcitabine and cisplatin alone without bevacizumab. A CT scan done post completion of 3 cycles of treatment showed response to therapy. The patient did not continue any further chemotherapy at this time due to toxicity issues. No PARP inhibitor was offered due to *BRCA* negative status on germline testing at the time. The patient developed progressive disease after 9 months and underwent a second surgery with excision of the vaginal vault and the pelvic mass and omentectomy. Histopathology was suggestive of metastatic HGSOC. For the ideal therapeutic options, the sample was sent for multi-analyte Exacta^®^ analysis. The overview of the treatment given is summarised in (Figure 1).

**Figure 2:**
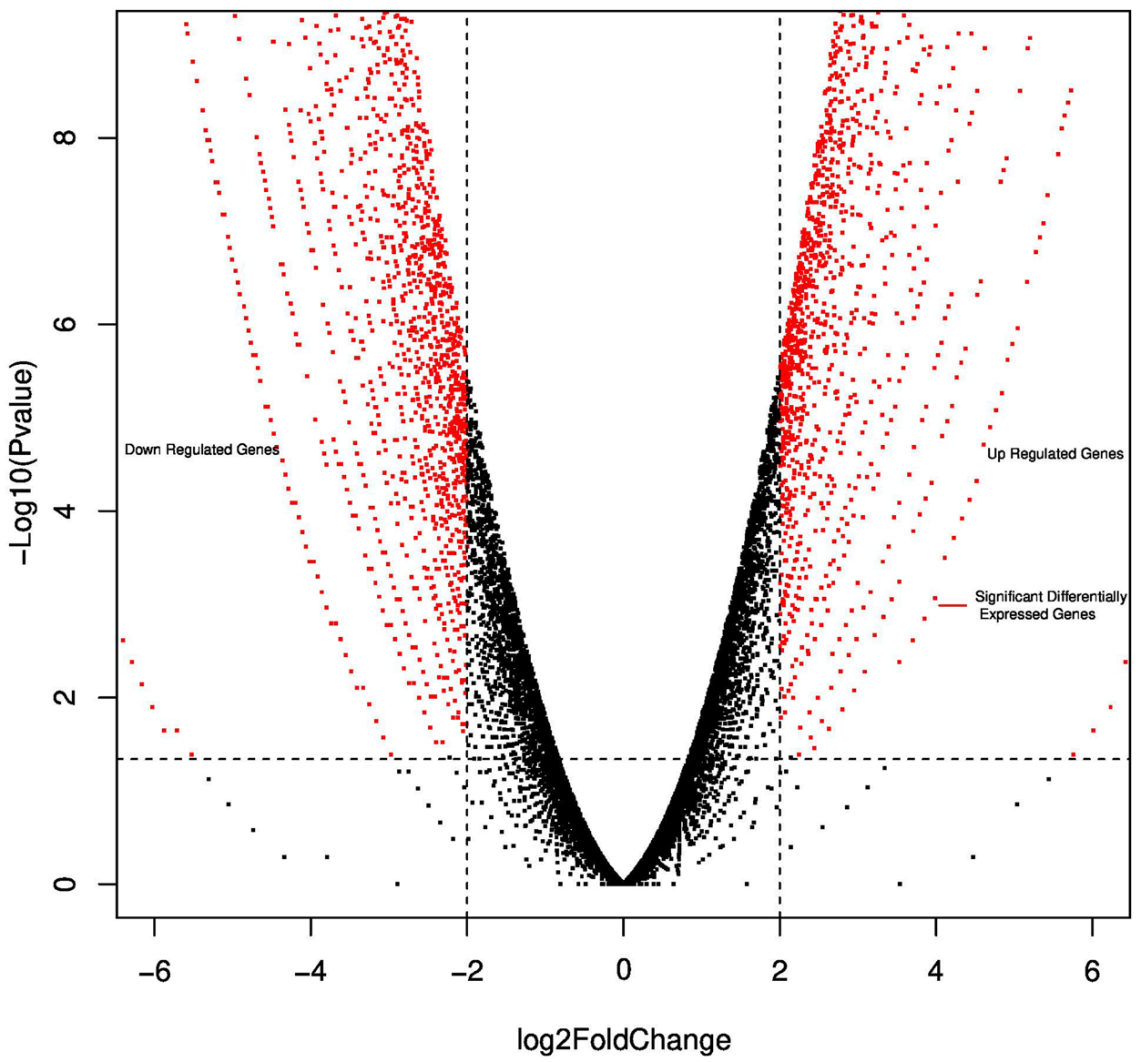
Volcano plot reporting *P* values against fold changes. The Volcano plot indicates -log 10 (*P*-value) for genes (Y-axis) plotted against their respective log 2 (fold change) (X-axis). The red dots represent significantly upregulated and downregulated Differentially Expressed Genes (DEG); black indicates no significant difference.

**Figure 3:**
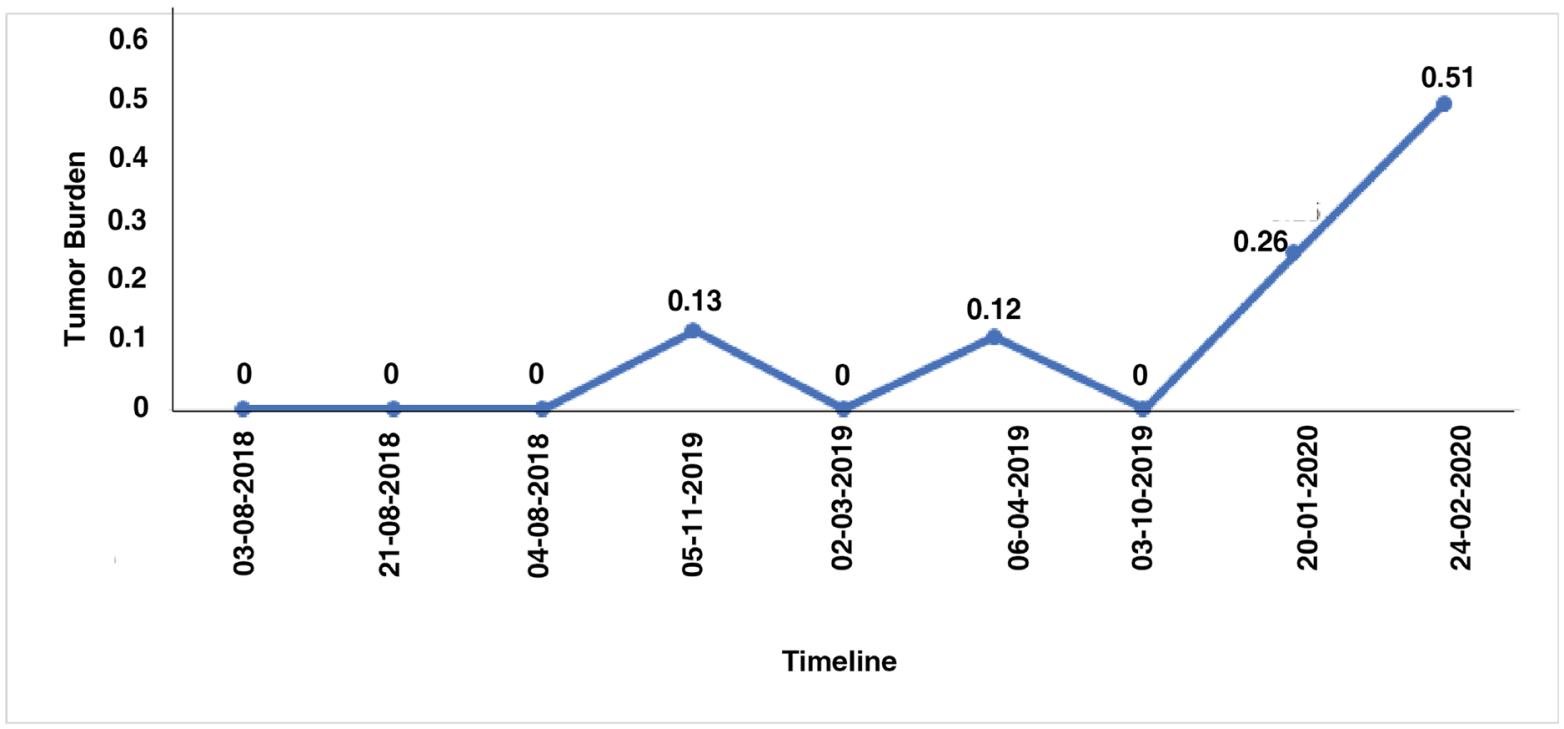
Longitudinal analysis of tumor mutation burden using the number of SNVs detected in each cell free DNA samples.

The integrative, multi-analyte Exacta^®^ test includes molecular analysis of comprehensive gene expression, DNA mutation profiling, chemosensitivity assay, immunohistochemistry and immunocytochemistry. For targeted transcriptome analysis, RNA from tumor tissue and adjacent normal tissue were used. Significant differential expressed genes were called using the following threshold: absolute log fold-change ≥ 2 and *p*-value < 0.05 (Figure 2). The gene expression analysis showed the expression of 6984 genes in which 1970 genes were differentially regulated when compared to adjacent normal tissue. Out of 1970 genes 957 genes were upregulated and 1013 genes were downregulated (Supplementary Table 1). Next generation sequencing analysis for mutations and amplifications of 409 oncogenes and tumor suppressor genes was carried out on the FFPE tumor tissue and cell free DNA. Tumor mutation analysis detected mutations in genes *CTNNB1* (p. G34V), *TP53* (p. N263fs), *PKHD1* (p. R3107Q) and *IGF2R* (p. A1425A) mutations. Copy number alteration analysis showed a copy loss in chromosomal regions including 4p, 4q, 5p, 5q, 6p, 7p, 10q, 11p, 12q, 13q, 17p, 17q, 18q, 19p and a copy gain in 7q and 16p. Longitudinal mutation profiling of cell-free DNA (cfDNA) was performed for the serial monitoring of circulating tumor burden in patient. Mutation load varied in the range 0% to 0.13% until 2019. However marginal increase in the mutational load of 0.26% and 0.56% was observed in the next follow-up samples (Figure 3). *TP53* (p. A159V and p. W146fs) gene mutation was observed in cell free nucleic acid analysis and are reported to be associated with early relapse and adverse prognosis in HGSOC. She was negative for any germline mutations.

**Figure 4:**
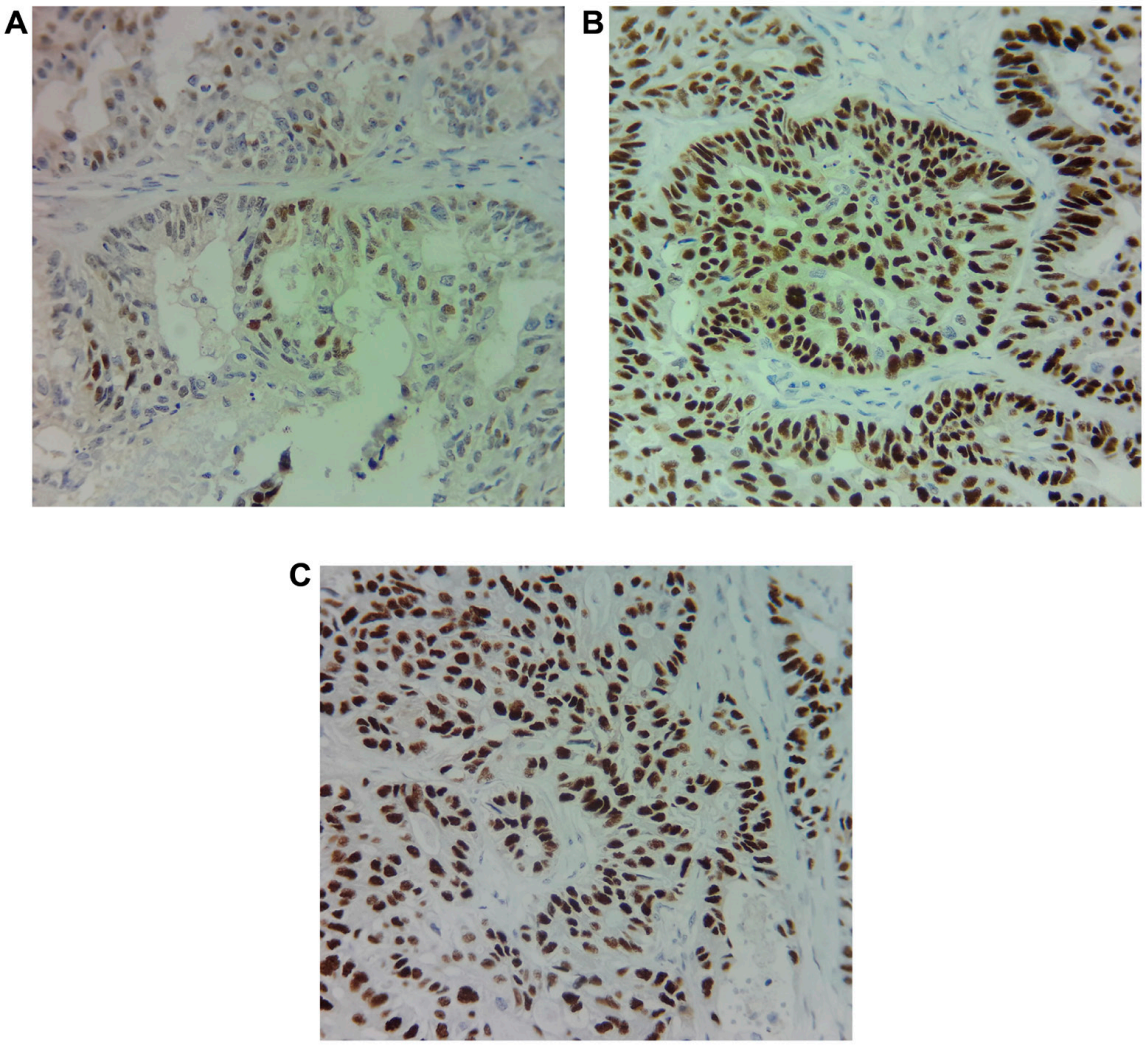
Immunohistochemical staining of (**A**) Androgen receptor (AR), (**B**) Estrogen receptor (ER) and (**C**) progesterone receptor (PR) shows 30%, 90% and 80% of expression respectively in the primary tumor cells.

The immunohistochemistry of the tissue samples revealed strong nuclear staining for ER (90% of tumor cells) and PR (80% of tumor cells); and moderate staining for AR (30% of tumor cells) (Figure 4). Chemosensitivity assay was performed on circulating epithelial cells (CECs) isolated from peripheral blood sample and showed high and modest response to vinorelbine and temsirolimus respectively.

Patient was then proposed treatment with Exacta^®^ based novel regimen with weekly injection of temsirolimus and oral therapy with tab bicalutamide 50 mg once daily. Temsirolimus was used due to the presence of PTEN loss (10q23.2) on genomic analysis and due to chemosensitivity analysis showing response *in vivo*. Bicalutamide was used due to the immunohistochemistry showing AR expression and also due to the fact that PTEN loss is known to confer resistance to aromatase inhibitor based therapy targeting the ER/PR. She was also started on supplementary treatment with quercetin and vitamin E capsules as per Exacta^®^ based recommendation. The patient received 3 cycles of the combination therapy. However, temsirolimus was stopped since she started experiencing vasomotor side effects and patient was continued on tab bicalutamide 50 mg once daily, quercetin and vitamin E. She remains with no evidence of disease and no disease relapse over the last 30 months of Exacta^®^ guided therapy.

## DISCUSSION

Ovarian cancer accounts for 295,414 new cases and 184,799 deaths around world-wide (GLOBOCAN 2018). More than 90% of the malignant cancers are of epithelial in origin and 70% of these malignant cancers belong to HGSOC [12]. The first line treatment for HGSOC includes surgery followed by combination chemotherapy. The patient presented in this study showed a relapse after the first line therapy. She developed recurrent disease two years later and since it was still considered to be platinum sensitive, the patient was treated with platinum-based combination therapy. Targeted therapy with bevacizumab a vascular endothelial growth factor (VEGF) inhibitor was added due to known added benefit in recurrent disease, however, had to be stopped due to toxicity [13]. No PARP inhibitor was offered due to *BRCA* negative status on germline testing at the time. It is known that patients with ovarian cancer who fail two lines of therapy have very poor prognosis. An exploratory multi-analyte testing was carried out at this juncture to understand the biology of this tumor better and in an attempt to find newer therapeutic options.

Mutational status of cfDNA reflects the genetic characterization of tumor lesion. *TP53* is the most frequently mutated tumour-suppressor gene in human cancer with the highest frequency of 80% in HGSOC [14]. Studies have highlighted the potential of cfDNA as diagnostic and prognostic tool for ovarian cancer and showed a relation between *TP53* mutations detected in cfDNA at diagnosis and residual disease or disease progression. Various *TP53* hotspot mutations can have different implications on outcome of disease and in response to chemotherapy [15]. Tumor mutation burden from cfDNA is used as an independent biomarker to assess the response to immunotherapy in many cancers [16, 17]. Elevated tumor mutation burden has shown prolonged response to immune check point inhibitors in platinum resistant ovarian cancer [18]. The present report shows marginal increase in the mutation load with long-term response to bicalutamide, an AR antagonist suggesting tumor mutation burden as biomarker to monitor the treatment response.

In the current study, we identified high expression of AR protein in the patient. AR, a steroid hormone receptor belongs to the nuclear receptor superfamily and is activated by androgen hormones. Although no specifics cut-offs are practiced, in breast cancer some studies have used at least 1% nuclear staining of any intensity (1+ to 3+) as a positive AR IHC assay [19]. In a Phase II study of single agent enzalutamide in AR positive recurrent ovarian cancer ≥ 5% AR expression by IHC was considered positive [20]. The activation of AR leads to its nuclear localization and regulation of expression of genes involved in different physiological and pathological functions [21]. AR signalling has been associated with tumorigenesis and metastasis of cancers such as prostate, bladder, kidney, lung, breast, liver and ovary. Androgens have been shown to involved in cell proliferation and invasion of ovarian cancer cells suggesting targeting AR as a capable treatment choice [21, 22]. AR targeted therapies have been widely employed in breast and prostate cancers [23–25]. Bicalutamide, an AR antagonist which binds to the AR block the action of androgens. It is widely used, especially in the Asia Pacific region as targeted therapy of prostate cancer and was used here in addition to temsirolimus [24, 26]. Temsirolimus was used due to PTEN loss seen in genomic analysis. PTEN loss is also known to confer resistance to aromatase inhibitor based therapy targeting the ER/PR. The response from bicalutamide, an AR inhibitor suggests that anti-androgen- based therapies could be an effective treatment option for AR positive ovarian cancer patients. Although the patient presented in this case showed relapse after the first line and second line therapy, the patient has now been without any relapse after being started on this Exacta^®^ guided treatment as a result of the AR directed therapy with bicalutamide. This response is not thought to be from temsirolimus since the patient was only able to take 3 cycles of the drug due to severe toxicity issues.

In conclusion, we present a patient with advanced HGSOC with multiple relapses with moderate AR expression detected on multi-analyte Exacta^®^ based analysis. The higher expression of AR in the patient provides strong evidence of pathogenicity. Further, targeting AR with bicalutamide based therapy resulted in a durable response in this patient. Our result suggests targeting AR using bicalutamide as an efficient treatment strategy in AR positive HGSOC.

## SUPPLEMENTARY MATERIALS




