# Addendum: A case report of androgen receptor inhibitor therapy in recurrent high-grade serous ovarian cancer

**DOI:** 10.18632/oncotarget.28262

**Published:** 2022-08-04

**Authors:** Sewanti Limaye, Prashant Kumar, Ramya Pragya, Janani Sambath, Darshana Patil, Ajay Srinivasan, Sachin Apurva, Navin Srivastava, Sanket Patil, Revati Patil, Vineet Datta, Dadasaheb Akolkar, Rajan Datar

**Affiliations:** ^1^Kokilaben Dhirubhai Ambani Hospital and Medical Research Institute, Mumbai, Maharashtra, India; ^2^Datar Cancer Genetics Limited, Nasik, Maharashtra, India; ^3^Institute of Bioinformatics, International Technology Park, Bangalore, Karnataka, India; ^4^Manipal Academy of Higher Education, Manipal, Karnataka, India

**Consent was added to this article:** The patient provided consent (by signing on a consent document) at the time of tumor tissue and blood sample collection stating willingness to:

- Provide the required sample for tumor molecular profiling.
- Research use of deidentified data including publication of (deidentified) findings.

Original article: Oncotarget. 2020; 11:4358–4363. 4358-4363. https://doi.org/10.18632/oncotarget.27809

