# Supplementary material for: A case report of androgen receptor inhibitor therapy in recurrent high-grade serous ovarian cancer
Source: Oncotarget. 2020 Nov 17;11(46):4358–63. doi: 10.18632/oncotarget.27809 (PMC7679039; doi:10.18632/oncotarget.27809)
Supplement: Supplementary file 1 [file oncotarget-11-4358-s001.pdf]

# A case report of androgen receptor inhibitor therapy in recurrent high-grade serous ovarian cancer

## SUPPLEMENTARY MATERIALS

**Supplementary Table 1: List of differentially expressed genes from targeted transcriptome analysis with log2FC > 2 and adjusted *p*-value < 0.05.** See Supplementary Table 1
